# Plasminogen Activators in Neurovascular and Neurodegenerative Disorders

**DOI:** 10.3390/ijms22094380

**Published:** 2021-04-22

**Authors:** Manuel Yepes, Yena Woo, Cynthia Martin-Jimenez

**Affiliations:** 1 Department of Neurology, Emory University, Atlanta, GA 30329, USA; 2 Neuropharmacology and Neurological Diseases Section, Yerkes National Primate Research Center, Atlanta, GA 30329, USA; yena.woo@emory.edu (Y.W.); cynthia.alexandra.martin-jimemez@emory.edu (C.M.-J.); 3 Department of Neurology, Veterans Affairs Medical Center, Decatur, GA 30329, USA

**Keywords:** tissue-type plasminogen activator (tPA), urokinase-type plasminogen activator (uPA), neurodegeneration

## Abstract

The neurovascular unit (NVU) is a dynamic structure assembled by endothelial cells surrounded by a basement membrane, pericytes, astrocytes, microglia and neurons. A carefully coordinated interplay between these cellular and non-cellular components is required to maintain normal neuronal function, and in line with these observations, a growing body of evidence has linked NVU dysfunction to neurodegeneration. Plasminogen activators catalyze the conversion of the zymogen plasminogen into the two-chain protease plasmin, which in turn triggers a plethora of physiological events including wound healing, angiogenesis, cell migration and inflammation. The last four decades of research have revealed that the two mammalian plasminogen activators, tissue-type plasminogen activator (tPA) and urokinase-type plasminogen activator (uPA), are pivotal regulators of NVU function during physiological and pathological conditions. Here, we will review the most relevant data on their expression and function in the NVU and their role in neurovascular and neurodegenerative disorders.

## 1. Introduction

**Figure 1:**
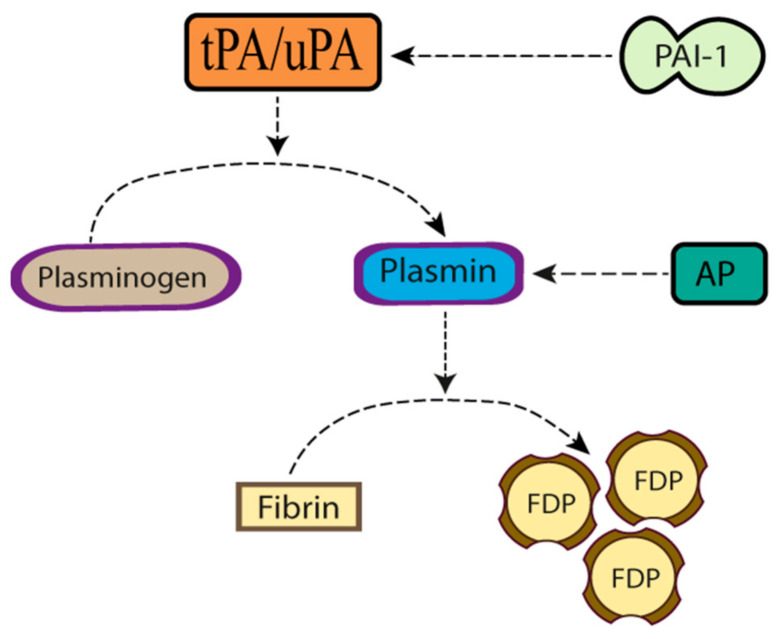
The plasminogen activating system. tPA: tissue-type plasminogen activator. uPA: urokinase-type plasminogen activator. AP: antiplasmin. PAI-1: plasminogen activator inhibitor-1. FDP: fibrin degradation products.

The plasminogen activation system is assembled by a cascade of proteases and their inhibitors that catalyze the conversion of the zymogen plasminogen into the two-chain protease plasmin (Figure 1). Plasminogen is a 90 kDa single-chain multidomain glycoprotein produced mainly in the liver [1] and assembled by 791 amino acids distributed in seven different structural domains: an N-terminal pre-activation peptide, 5 kringle domains and a C-terminal trypsin-like serine protease domain that harbors the catalytic triad His603, Asp646 and Ser741 [2]. Plasminogen binds to a plethora of highly heterogenous receptors on the cell surface, and this interaction not only triggers the generation of plasmin, but also activates cell signaling pathways that orchestrate a wide variety of functions including wound healing, angiogenesis, cell migration and inflammation [3]. Cleavage of plasminogen at the Arg561–Val562 bond by one of the two main plasminogen activators [tissue-type plasminogen activator (tPA) and urokinase-type plasminogen activator (uPA)] generates a two-chain plasmin molecule assembled by an N-terminal heavy-chain and a disulfide-linked C-terminal light chain containing the proteolytically active site. Importantly, the conversion of plasminogen into plasmin is enhanced when plasminogen is bound to fibrin or to the cell surface [4]. The generation of plasmin is tightly controlled at different steps of the plasminogen activating system. Accordingly, while plasminogen activator inhibitor-1 (PAI-1) and plasminogen activator inhibitor-2 (PAI-2) antagonize the proteolytic effect of tPA and uPA [5], α_2_-antiplasmin inhibits plasmin. In the intravascular space, plasmin acts not only as an effector of the fibrinolytic system by degrading fibrin, but also as an immune regulator [6]. Likewise, on the cell surface plasmin triggers the degradation of multiple components of the extracellular matrix (ECM) and basement membrane, including collagen, vitronectin, laminin, fibronectin and proteoglycans.

## 2. The Neurovascular Unit

**Figure 2:**
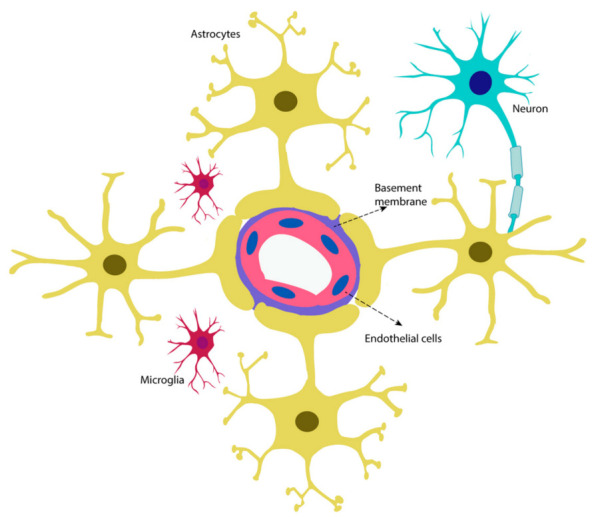
The neurovascular unit. Schematic representation of the cellular and non-cellular components of the neurovascular unit.

The concept of the neurovascular unit (NVU) describes a dynamic interaction in the central nervous system between endothelial cells ensheathed by a basement membrane, and surrounding pericytes, astrocytes, microglia and neurons (Figure 2). The nature of the interplay between these cellular and non-cellular components has led to the proposal that the NVU is a single functioning unit responsible for the maintenance of cerebral hemostasis [7].

## 3. Plasminogen Activators in the Neurovascular Unit under Physiological Conditions

### 3.1. Tissue-Type Plasminogen Activator

Tissue-type plasminogen activator (tPA) is a 70-kDa serine proteinase secreted as a single-chain form (that upon its cleavage by plasmin at Arg275-Ile276 generates an active two-chain form held together by disulfide bonds). The tPA molecule is assembled by four domains: an amino-terminal region (fibronectin-like or finger domain), an EGF-like domain, two kringles and one serine protease region that harbors the active site residues His322, Asp371 and Ser478 [8]. In the neurovascular unit (NVU), tPA is found in endothelial cells, perivascular astrocytes, microglia, pericytes and neurons [9,10].

### 3.2. Tissue-Type Plasminogen Activator in the Neurovascular Unit 

#### 3.2.1. Cerebral Endothelial Cells

In endothelial cells tPA is stored in Weibel–Palade bodies, the specialized endothelial storage granules for von Willebrand factor [11]. The expression of tPA is increased at the transcriptional level in endothelial cells by a variety of stimuli, including vascular endothelial growth factor (VEGF), fluid shear stress, thrombin and histamine [12,13,14]. In turn, its release from a preformed pool is triggered by physical activity, β-adrenergic drugs, cholinergic agents, disseminated intravascular coagulation and hypoxia [15,16]. Studies with a primary monoclonal antibody that detected free and PAI-1-complexed human tPA revealed that in the non-human primate brain, tPA is found in a reduced number of endothelial cells of the microvasculature, most of them pre-capillary arterioles and post-capillary veins [17]. However, despite the relevance of these data, it is important to consider that since this report was published almost 3 decades ago no further effort has been made to characterize the expression of tPA with newer antibodies in endothelial cells of the brain. Finally, although tPA has been detected in in vitro lines of human microvascular endothelial cells [18], no in vivo studies have been published describing the expression of tPA in blood vessels of the human brain. 

#### 3.2.2. Pericytes

Very few studies have assessed the expression and function of tPA in pericytes. However, the few that have been published to this date indicate that zinc prompts the release of tPA from these cells [19], and that pericytes negatively regulate fibrinolysis-triggered endothelial cell-derived tPA [20].

#### 3.2.3. Astrocytes

**Figure 3:**
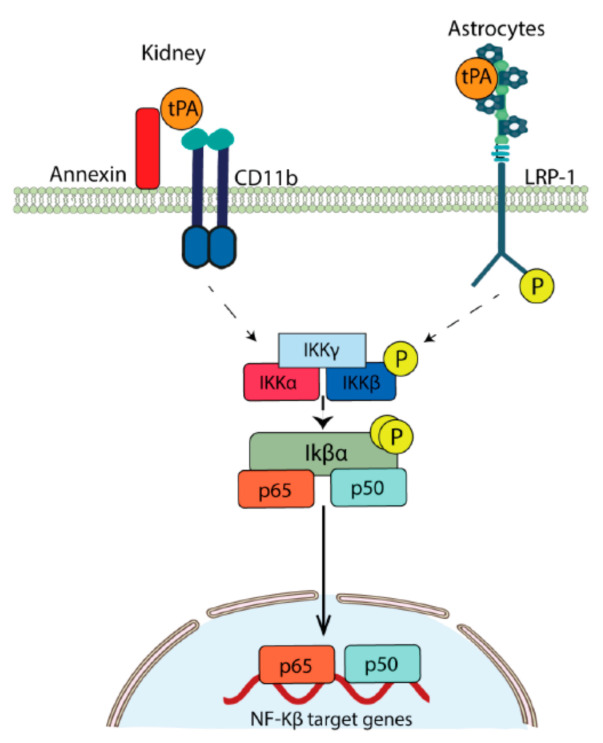
Mechanisms of tPA-induced NF-κB activation. Representative diagram of the proposed mechanisms whereby tPA activates the NF-κB pathway in the kidney and cerebral cortical astrocytes. In both cases, IKBα phosphorylation is followed by the nuclear translocation of p65/p50.

tPA is abundantly found in astrocytes, and several stimuli including hypoxia [21] and mechanical injury [22] trigger its release, both in vivo and in vitro. In line with these observations, tPA activates the NF-κB pathway in astrocytes [23], and its release into the basement membrane increases the permeability of the NVU [21]. The mechanism whereby tPA activates the NF-κB pathway is not completely understood. However, work with cerebral cortical astrocytes and rat kidney interstitial fibroblasts (NRK-49F) [24] revealed that the interaction between tPA and LRP1 triggers the phosphorylation of IKKα, which then allows p65/p50 to translocate to the nucleus [25]. Further work with kidney cells has shown that another mechanism whereby tPA activates the NF-κB is by triggering annexin 2-mediated aggregation of the integrin CD11B, which in turn prompts the phosphorylation of IKβ with the resultant nuclear translocation of p65/p50 [26] (Figure 3). In addition to its proinflammatory effect, an increase in the expression and activity of astrocytic tPA induced by multipotent mesenchymal stromal cells has been associated with neurite growth [27]. Furthermore, it has been proposed that astrocytes recycle tPA released in the synaptic cleft in response to glutamatergic signals [28], and that tPA released from astrocytes modulates the microglial response to endotoxins [29]. Together, these data indicate that the release of astrocytic tPA plays a pivotal role in the NVU as a regulator of the permeability of the astrocyte–basement membrane–endothelial cell interface, synaptic transmission, neuroinflammation and microglial function.

#### 3.2.4. tPA in Microglia

Inasmuch as a functional link between tPA and microglial activation has been experimentally demonstrated, it is not clear if microglia release tPA. However, it has been proposed that injured neurons release tPA, and that this tPA triggers the release of microglial tPA, which in turn causes neurodegeneration [30]. Independently of these considerations, experimental evidence indicates that tPA mediates endotoxin- and kainic acid-induced microglial activation via an annexin II-mediated mechanism [31] that does not require plasmin generation [32] and triggers neuronal apoptosis [33].

#### 3.2.5. Neuronal tPA

Neurons are a major reservoir of tPA in the central nervous system (CNS), and the release of neuronal tPA in the developing and mature brain plays a central role in the regulation of synaptic function and the response of the CNS to a variety of injuries. Indeed, the release of tPA by neuronal growth cones in the developing brain [34] induces neuronal migration [35] and neurite outgrowth and remodeling [36]. Noticeably, a similar sequence of events in the mature brain promotes neuronal recovery following an ischemic injury [37,38]. In contrast with the developing CNS, in situ zymography studies have revealed that only well-defined areas of the mature brain exhibit tPA-catalyzed proteolytic activity, namely the hippocampus, hypothalamus, thalamus, amygdala, cerebellum and meningeal blood vessels [39]. Furthermore, the interaction of tPA with *N*-methyl-D-aspartate (NMDA) receptors in these structures [40] regulates glutamatergic neurotransmission [41] and promotes the development of synaptic plasticity, as demonstrated in in vitro and in vivo models of long-term potentiation [42], learning [43,44], stress-induced anxiety [45] and visual cortex plasticity [37].

### 3.3. Urokinase-Type Plasminogen Activator

Urokinase-type plasminogen activator (uPA) is a 53 kDa serine proteinase secreted as a single-chain uPA (scuPA) with 411 amino acids assembled into three domains: an N-terminal domain homologous to the epidermal growth factor (responsible for its binding to uPAR), a kringle domain that interacts with plasminogen activator inhibitor-1 (PAI-1) and a C-terminal catalytic domain that harbors the active site with the His204, Asp255 and Ser356 amino acids triad [46]. The binding of scuPA to its receptor (uPAR) triggers its cleavage at K158-I159, thus prompting its conversion into an active two-chain form (tcuPA), with an A chain with the epidermal growth factor and kringle domains, and a B chain with the proteolytic domain [47]. In turn, tcuPA catalyzes the conversion of plasminogen into plasmin on the cell surface [48].

The receptor for uPA (uPAR) has 270 amino acids assembled into three cysteine-rich Cd59-like sequence domains (D1, D2 and D3) connected by short linker regions and bound to the surface of the plasma membrane by a glycosyl phosphatidylinositol (GPI) tail. Regulation of uPAR is accomplished by either an inactivating uPA-induced cleavage of the receptor between D1 and D2, or by endocytosis of a PAI-1–uPA–uPAR low-density lipoprotein receptor-related protein-1 (LRP1) complex assembled on the cell surface, which then recycles free uPAR back to the membrane to bind to more uPA [49].

### 3.4. Urokinase-Type Plasminogen Activator in the Neurovascular Unit (NVU)

#### 3.4.1. Cerebral Endothelial Cells

A substantial body of experimental evidence indicates that uPA and uPAR are found in endothelial cells [50], and that the release of this uPA and the increase in the expression of uPAR in endothelial cells triggered by a variety of stimuli [51,52] induces cell migration, angiogenesis [53] and capillary branching. However, it is important to take into account that most of these studies have been performed with cell lines, and that very few studies have assessed the in vivo expression of uPA in cerebral endothelial cells. With that in mind, it has been reported that *Cryptococcus neoformans* increases the expression of uPA in cerebral endothelial cells [54], and that microvascular endothelial cells upregulate uPA following an ischemic injury to the spinal cord in vivo [55].

#### 3.4.2. Astrocytes and Microglia

The abundance of uPA and uPAR is increased in glial cell tumors, particularly glioblastoma multiforme, where they have been reported to play a role in tumor growth [56]. In contrast, the role of astrocytic uPA and uPAR under physiological conditions is less well understood. However, recent studies indicate that the release of uPA under physiological conditions triggers astrocytic activation [57] and induces the formation of peripheral astrocytic processes [58]. The expression and role of microglial uPA and uPAR under non-pathological conditions is largely unknown, although in vitro studies have shown that endotoxins, kainic acid and neurogeneration increase their abundance in microglia [59].

#### 3.4.3. Neurons

Despite the fact that uPA and uPAR are abundantly found in developing neurons [60,61,62], their expression changes dramatically over a few days. Hence, while day in vitro (DIV) 3 neurons harbor uPAR in their cell body and neurites, at DIV 7 the expression of this receptor shifts to the axon shaft and growth cones, and at DIV 16 is restricted to the distal segment of some axons and very few growth cones [60]. Significantly, uPA/uPAR binding during the early stages of development induces neuritogenesis and neuronal migration via a plethora of mechanisms that do not always require plasmin generation [63,64]. More specifically, by promoting activation of integrins and the focal kinase adhesion (FAK) pathway, uPAR regulates the reorganization of the cytoskeleton [63], thus triggering axonal growth, neuronal migration [65] and dendritic branching [66]. In line with these observations, uPAR seems to be pivotal for the formation of neuronal circuits that underlie the development of language and cognition, and dysregulation of the uPA/uPAR system has been linked to epilepsy and cognitive and developmental disorders [67].

In contrast, the expression and role of uPA/uPAR in the mature brain have been less studied. However, recent studies with human, murine and non-human primate brains indicate that uPA is abundantly found in synapses of the second and fifth cortical layers of the cerebral cortex, and that uPA/uPAR binding modulates excitatory neurotransmission by regulating the synaptic expression of neuronal cadherin (NCAD) [68]. Additionally, these studies showed that uPA induces the expression of NCAD in the synapse, and that the resultant generation of NCAD-dimers leads to the formation of synaptic contacts in neurons maintained under physiological conditions [68].

## 4. Plasminogen Activators in the Neurovascular Unit (NVU) under Ischemic Conditions

Ischemic stroke is a leading cause of mortality and disability in the world [69]. Significantly, plasminogen activators are pivotal for the protection and repair of the NVU that has suffered an ischemic injury. Indeed, while acute cerebral ischemia causes the rapid release of tPA from each cellular compartment of the NVU [70], the abundance and activity of uPA increase only during the recovery stages from the ischemic insult [61]. These data have led to the proposal that while the early release of tPA restores the patency of the occluded blood vessel and has a neuroprotective effect, the delayed release of uPA promotes the repair of the damaged NVU. Below we will review data on the role of tPA and uPA in each component of the NVU under hypoxic/ischemic conditions.

### 4.1. Endothelial Cells

#### 4.1.1. Tissue-Type Plasminogen Activator

The effect of hypoxia on the release of tPA from human cerebrovascular endothelial cells has been poorly characterized. However, studies with human saphenous and umbilical veins [71,72] have shown that hypoxia decreases the abundance and activity of tPA in endothelial cells, and that this effect is accompanied by an increase in the expression and activity of PAI-1. Furthermore, in vitro studies with rat brain microvascular endothelial cells indicate that tPA has a harmful effect on endothelial cells exposed to oxygen and glucose deprivation conditions [4,73]. In contrast with these in vitro studies, in vivo observations have revealed an increase in the concentrations of tPA and PAI-1 in the intravascular space of patients suffering an acute ischemic stroke [74], suggesting that endothelial cells release tPA into the intravascular space as an attempt to restore the patency of the occluded blood vessel. This is supported by the observation of complete or nearly complete recovery of neurological function in acute ischemic stroke patients intravascularly treated with recombinant tPA within 3–4.5 h of the onset of symptoms [75,76].

A growing body of experimental evidence indicates a link between plasminogen activation and the immune system. Indeed, while some studies have revealed that plasminogen activators play a role in bradykinin-mediated endothelial cell activation [77], others show that an interaction between plasmin and factor XII increases the permeability of the neurovascular unit in neurodegenerative diseases [78,79]. Importantly, in apparent discrepancy with a proinflammatory role of plasmin, in vivo studies with an animal model of cerebral ischemia suggest that tPA attenuates the activation of the immune response in the neurovascular unit that has suffered an ischemic injury [80].

#### 4.1.2. Urokinase-Type Plasminogen Activator

The role of uPA in hypoxic/ischemic cerebral endothelial cells is even less well studied. Indeed, although studies with human umbilical endothelial cells (HUVEC) [81] and human microvascular endothelial cells [82] have revealed that a hypoxia-induced, hypoxia-inducible factor (HIF)-mediated increase in uPAR expression in endothelial cells triggers angiogenesis and cell migration [83], the effect of hypoxia on uPA and uPAR expression and function in cerebral endothelial cells has not been characterized. Independently of these considerations, clinical studies indicate that the intravascular administration of recombinant uPA effectively restores the patency of the occluded blood vessel and improves neurological outcome in acute ischemic stroke patients [84,85].

### 4.2. Astrocytes

#### 4.2.1. Tissue-Type Plasminogen Activator

As discussed above, tPA is abundantly found in astrocytes [21], and the release of astrocytic tPA has a direct effect on the permeability of the NVU. Indeed, the interaction between tPA, released from perivascular astrocytes in response to the ischemic injury, and the low-density lipoprotein receptor-related protein-1 (LRP-1) found in astrocytic end-feet processes, activates an NF-κ-regulated inflammatory response [86] and triggers the detachment of perivascular astrocytes from the basement membrane, which in turn increases the permeability of the blood–brain barrier, thus causing ischemic cerebral edema [21]. In line with these observations, the intracerebroventricular administration of recombinant tPA induces an LRP-1-mediated increase in the permeability of the NVU [87]. The translational relevance of these observations is underscored by neuroradiological studies showing that the intravenous administration of recombinant tPA to acute ischemic stroke patients also increases the permeability of the blood–brain barrier [88] which is in line with a reported 10-fold increase in the risk of hemorrhagic complications in recombinant tPA (rtPA)-treated stroke patients [75]. Interestingly, besides its effect on the permeability of the NVU, experimental evidence indicates that multipotent mesenchymal stromal cell-induced tPA activity in astrocytes promotes neurorepair after stroke by facilitating neurite outgrowth in the ischemic area [27,89].

#### 4.2.2. Urokinase-Type Plasminogen Activator

The roles of astrocytic uPA and uPAR in the ischemic brain have only recently been studied. Accordingly, it was reported that the binding of uPA released from neurons to uPAR recruited to the astrocytic plasma membrane in response to the ischemic injury, induces astrocytic activation [57]. Ezrin is a protein that regulates the reorganization of the actin cytoskeleton [90] and the formation of microvilli, filopodia and lamellipodia [91]. In the cytosol, ezrin exists in an inactive conformation. However, following its recruitment to the plasma membrane, ezrin is activated by phosphorylation at a conserved Thr567 residue [92]. Ezrin is abundantly found in astrocytic filopodia [93], and its activation is required for the formation of peripheral astrocytic processes [94]. Significantly, uPA induces the expression of ezrin in astrocytes, thus triggering the formation of peripheral astrocytic processes that, upon embracing the pre- and post-synaptic compartments, preserve the integrity of the tripartite synapse that has suffered an ischemic insult [58].

### 4.3. Microglia

#### 4.3.1. Tissue-Type Plasminogen Activator

Microglial activation is a key step in a sequence of events that trigger not only cell death but also neurorepair in the ischemic brain [95]. Remarkably, tPA is pivotal for microglial activation [31], and in support of these observations, genetic deficiency of tPA attenuates cerebral ischemia-induced microglial activation [32]. Interestingly, the N-terminal fibronectin type III finger domain of tPA also mediates endotoxin-induced microglial activation, most likely by its interaction with annexin II on the cell membrane [96]. Further work has revealed that LRP-1 mediates the effect of tPA on microglial activation in the ischemic brain [97], and that the resultant downstream activation of latent platelet-derived growth factor-CC (PDGF-CC) increases the permeability of the NVU [98]. Additionally, it was reported that by modulating the release of cytokines, interferon-β attenuated the effect of tPA-induced microglial activation on the permeability of the NVU [99].

#### 4.3.2. Urokinase-Type Plasminogen Activator

It has been recognized that cultured human microglia express uPAR [100], and that the abundance of this receptor in microglia is greatly increased by treatment with endotoxins. More importantly, experimental studies have shown that uPAR is able to induce microglial activation by a mechanism that always requires uPA [101], but that in some cell lines is mediated by MMP-9 [102]. Strikingly, despite the importance of these observations, the role of uPA/uPAR in cerebral ischemia-induced microglial activation is still poorly understood.

### 4.4. Neurons

#### 4.4.1. Tissue-Type Plasminogen Activator

Despite the fact that a large number of studies agree on the fact that hypoxia and ischemia trigger the release of neuronal tPA [70,103,104,105], there is significant disagreement on the effect that this tPA has on cell survival. Indeed, results from early studies showing that mice genetically deficient in tPA (tPA^−/−^) have a significant decrease in the volume of the ischemic lesion following transient occlusion of the middle cerebral artery (tMCAo) [104,106] seeded the idea that tPA has a neurotoxic effect in the ischemic brain. Strikingly, this idea lingered for a long time despite subsequent publications by other groups describing an increase in the volume of the ischemic lesion in tPA^-/-^ mice [106], and either a beneficial [107] or even a lack of effect [108] of rtPA treatment on the volume of the ischemic lesion following tMCAo.

This discrepancy was dramatically brought to the forefront of the scientific discussion by the publication of a National Institute of Neurological Disorders and Stroke (NINDS)-led clinical study showing that treatment with recombinant tPA within 3 h of the onset of symptoms was associated with complete or nearly complete recovery in neurological function in a significant number of acute ischemic stroke patients [75,109], and by the subsequent incorporation of rtPA in the protocols used for the treatment of these patients [110]. Notably, although treatment with rtPA also increases the risk of intracerebral hemorrhage [109] and augments the permeability of the NVU [89], to this date no clinical study has shown a neurotoxic effect caused by rtPA treatment. The translational impact of this disagreement between basic and clinical researchers has been heightened by the observation that, following its intravenous administration, rtPA crosses through the blood–brain barrier and permeates the ischemic tissue [111]. In other words, if findings published by basic researchers are true, then clinicians are treating acute ischemic stroke patients with a neurotoxic agent. For obvious reasons this discrepancy needs to be resolved as it has called into question the clinical translatability of basic science research.

Early studies proposed that tPA mediated excitotoxin-induced neuronal death, which is a pivotal mechanism of cell death in the ischemic brain. Indeed, it was found that genetic deficiency of tPA attenuated kainic acid-induced hippocampal cell death [112] via plasmin-induced proteolysis of laminin in the extracellular matrix [113], and that tPA^-/-^ mice were resistant to KA-induced seizures [112]. This study was followed by work from a different group of researchers that measured the volume of the ischemic lesion in rodents injected with NMDA into the striatum and then intravascularly treated with 10 mg/Kg/IV of rtPA [114]. These investigators found that rtPA treatment enhanced the harmful excitotoxic effect of NMDA, which was interpreted as another demonstration of a neurotoxic effect of tPA. In contrast, a different group of investigators using a similar experimental paradigm but a different dose of rtPA (0.9 mg/Kg/IV, the same dose used to treat acute ischemic stroke patients), found an opposite effect: a decrease in the volume of the necrotic lesion in rtPA-treated animals [115]. Furthermore, they also found that the damage induced by the intrastrial injection of NMDA was significantly attenuated in mice overexpressing tPA only in neurons. Additionally, it was soon clear that the intracerebral injection of an excitotoxin (kainic acid) caused a transient increase in the activity of tPA in cells of the hippocampal CA1 layer that survived the excitotoxic injury [116], and this was followed by a report indicating that tPA protected hippocampal cells from the harmful effects of the excitotoxic injury [117].

This led a different group of investigators to quantify neuronal survival in cerebral cortical neurons incubated with NMDA in the presence of 0–500 nM of either proteolytically active tPA or a mutant of tPA with an alanine for serine substitution at the active site Ser481 that rendered it unable to catalyze the conversion of plasminogen into plasmin (proteolytically inactive tPA) [115]. These experiments revealed that tPA caused a modest increase in NMDA-induced neuronal death only at doses greater than 100 nM, which are not found in in vivo systems, even after the intravenous administration of rtPA. Furthermore, it was discovered that at concentrations found in the ischemic brain, tPA attenuated NMDA-induced neuronal death by a mechanism that did not entail plasmin generation but required the co-receptor function of a member of the low-density lipoprotein receptor (LDLR) family, most likely LRP1. In an attempt to explain these discrepancies, it was proposed that selective activation of NMDA receptors by single-chain but not two-chain tPA is responsible for the neurotoxic effect of tPA [118], and therefore that treatment with two-chain tPA is more efficient than single-chain tPA to reduce the volume of the ischemic lesion and promote functional recovery after the experimental induction of an ischemic stroke [119]. Together, these results show that a causal link between tPA and cerebral ischemia- and excitotoxin-induced neuronal death was difficult to establish, as it seemed to depend on the chemical structure and dose of rtPA as well as the specific experimental paradigm used in each report.

The resultant renewed interest of the scientific community to understand the role of neuronal tPA in the ischemic brain led a group of investigators using an in vitro model of oxygen and glucose deprivation (OGD) to discover that treatment with 5 nM of rtPA prevented cell death in cerebral cortical neurons exposed to 55 min of OGD conditions, and that this effect was mediated by LRP1 and open synaptic NMDA receptors [104]. Remarkably, the detection of a maximal neuroprotective effect within the first three hours after OGD bears a notable resemblance with the maximal neurological recovery observed in acute ischemic stroke patients treated with rtPA within three hours of the onset of symptoms [75]. The obvious lack of a clot in this in vitro system indicated that a mechanism other than thrombolysis mediates tPA’s neuroprotective effect, and this possibility was confirmed by the finding that treatment with recombinant tPA after tMCAo also decreased the volume of the ischemic lesion in animals genetically deficient in plasminogen (Plg^-/-^). These data indicate that tPA has a neuroprotective effect in the ischemic brain that is not mediated by the generation of plasmin and instead requires the co-receptor function of the NMDAR and a member of the LDLR family.

#### 4.4.2. Urokinase-Type Plasminogen Activator

The role of uPA in the ischemic NVU is less well understood. Indeed, early studies with an animal model of permanent cerebral ischemia induced by occluding a distal branch of the middle cerebral artery with a surgical suture showed a decrease in the volume of the ischemic lesion in mice genetically deficient in uPAR [105] but not uPA [120]. Interestingly, using a similar animal model of cerebral ischemia, a different group of investigators detected a large increase in uPA-catalyzed proteolysis 72 h after the onset of the ischemic injury [121]. This was followed by the observation that the concentrations of uPA in the culture medium of cerebral cortical neurons remained unchanged during 60 min of exposure to OGD conditions [61].

However, in an unexpected turn of events, it was found that these neurons released large amounts of uPA after they were returned to normoxic conditions. Importantly, this uPA did not seem to have an effect on cell death, as there was no difference in neuronal survival between Wt and uPA^-/-^ neurons exposed to OGD conditions [61]. The in vivo significance of these observations was supported by the finding that although cerebral ischemia did not have an effect on the abundance of uPA during the acute phase of the ischemic injury, the expression of uPA in the ischemic tissue increased during the recovery period.

The finding that the delayed release of uPA following a hypoxic/ischemic injury did not have an effect on neuronal survival or the volume of the ischemic lesion, led researchers to investigate if uPA plays a role in neurorepair. Noticeably, this possibility was supported by the observation that compared to wild-type (Wt) littermate controls, uPA^-/-^ and uPAR^-/-^ mice had a protracted recovery in neurological function following tMCAo, and that treatment with ruPA or the release of endogenous uPA prompted functional recovery in Wt and uPA^-/-^, but not in uPAR^-/-^ mice [60,61].

Further studies showed that the release of uPA promoted the recovery of axonal boutons and post-synaptic terminals disassembled by the ischemic injury. More specifically, it was found that by regulating the expression and activity of GAP-43, neuronal uPA promoted the regeneration of axons damaged by the ischemic injury [122]. Furthermore, by its ability to regulate the expression of ezrin, uPA was able to reorganize the cytoskeleton of the post-synaptic density, prompting the recovery of dendritic spines that disappeared in the earlier stages of the ischemic insult [61]. In line with these observations, in vivo studies indicated that intravenous treatment with recombinant uPA 24 h after the onset of the ischemic injury increased the number of synaptic contacts in the area that surrounds the necrotic core [57].

In summary, the data available to this date indicate that the expression of uPA and uPAR increases in the recovery stages of an ischemic stroke, and suggest that uPA binding to uPAR plays a central role in the process of neurorepair following an acute ischemic injury. These observations are supported by reports from other groups indicating that uPAR modulates peripheral nerve regeneration after a crushed nerve [67], and that genetic deficiency of uPA aggravates the motor deficit and increases neuronal death in an animal model of traumatic brain injury [121].

## 5. Plasminogen Activators in Neurodegenerative Disorders

The concept of neurodegenerative disorders encompasses several clinical entities including Alzheimer’s disease (AD), Parkinson’s disease (PD) and amyotrophic lateral sclerosis (ALS), all characterized by the progressive decline of neuronal function. Remarkably, a rapidly growing knowledge of the pathophysiology of these disorders has led to two important conclusions. First, that they are not caused by isolated neuronal pathology, but instead that a dysfunctional NVU is a contributory factor in many of them [122,123]; and second, that a dysfunctional plasminogen activating system plays a still poorly understood role in their pathophysiology. Together, the data reviewed below underscore the relevance of the interaction between the plasminogen activating system and neurodegeneration, and how research on this interaction may unveil potential targets for the development of strategies for their prevention and treatment. 

### 5.1. Plasminogen Activators in Alzheimer’s Disease 

AD affects approximately 46.8 million people worldwide, and this number is expected to reach 131.5 million by 2050 [124]. It is a dual proteinopathy, that accounts for almost 60–80% of all dementias, and is characterized by the extracellular deposition of Aβ 1–40 and 1–42 fibrils in neuritic plaques and intracellular aggregates of hyperphosphorylated tau in neurofibrillary tangles (NFT). Importantly, a substantial number of studies have found that even in the early stages of this disease the NVU is dysfunctional. Accordingly, a long-time accepted neurocentric theory of the genesis of AD has slowly been integrated into a more holistic model that includes all the cellular and non-cellular components of the NVU.

### 5.2. Endothelial Cells

There is ample evidence implicating endothelial cell dysfunction in the pathophysiology of AD. Indeed, virtually all AD patients exhibit endothelial cell degeneration and abnormal thickening of the perivascular basement membrane in zones with Aβ deposition [125]. These morphological changes underlie the reduction in cerebral blood flow, and impaired cerebrovascular reactivity and neurovascular coupling observed even in early stages of the disease [126,127]. Importantly, the few studies published to this date on plasminogen activators and endothelial cells in AD indicate that although Aβ does not have an effect on the release of endothelial tPA [19], deficiency of this plasminogen activator, likely caused by increased PAI-1, underlies the impairment in neurovascular coupling observed in mice expressing the Swedish mutation of the amyloid precursor protein (APP; tg2576) [128]. It has also been postulated that plasminogen derived from the intravascular space causes an inflammatory response and Aβ deposition. More specifically, it has been reported that depletion of plasminogen in the intravascular space attenuates microglial activation and improves AD pathology in mice transgenic for human APP/Presinilin 1 with five early-onset familial AD mutations [78]. In contrast with these studies, the role of uPA in endothelial cell dysfunction in AD has been addressed by fewer investigators. However, it has been reported that Aβ induces the expression of uPA in cultured human cerebrovascular smooth muscle cells [129], and that LRP1 in endothelial cells regulates the efflux of Aβ into the intravascular space [130]. The translational relevance of these observations, performed in the murine brain, should be understood in the light of studies indicating that human brain endothelial cells do not express LRP1 [131].

### 5.3. Astrocytes

Several studies have reported an association between early astrocytic activation [132] and poor prognosis in advanced phases of this disease [133]. Interestingly, this process seems to affect only a sub-population of astrocytes with an increased abundance of aquaporin-4 in their end-feet processes [134]. This is of special interest, because the interaction between astrocytic end-feet processes and endothelial cells modulates the permeability of the NVU [135]. In line with these observations, in vitro and in vivo studies have revealed an increase in the permeability of the NVU in different animal models of AD [129] and in the brain of AD patients [136]. Finally, astrocytes are tightly associated with Aβ catabolism, and these cells display an abnormal response upon exposure to Aβ [137]. Strikingly, no studies have directly addressed the specific role of astrocytic tPA and uPA in the pathogenesis of Alzheimer’s disease.

### 5.4. Microglia

Microglial activation in the brain of AD patients [138] has been linked to the triggering of a neuroinflammatory response that promotes Aβ-containing plaque formation and neuronal degeneration [139]. However, more recent studies have revealed that microglial activation in AD is a heterogenous process, and in line with these observations, specific and well-differentiated subpopulations of microglia also seem to have a protective effect in the brain of AD patients [140]. Furthermore, it has been reported that the plasminogen activating system modulates the activation of microglia in AD [141] and that treatment with rtPA triggers the activation of the above-mentioned neuroprotective microglial phenotype [142]. In contrast, the role of uPA in microglial activation in AD has been less well studied. However, it has been reported that Aβ-treated human microglia upregulate uPA and uPAR [143] and that uPAR is a marker of microglial activation in the brain of AD patients [144].

### 5.5. Neurons

The amyloid hypothesis of AD is a neurocentric model in which Aβ deposition leads to progressive tau hyperphosphorylation, synaptic dysfunction and neuronal loss. However, despite its importance and long-time acceptance, a growing body of experimental evidence suggests that not only neurons, but all cellular and non-cellular elements of the NVU, play key roles in the pathogenesis of this disease [145]. Independently of these considerations, knowledge gathered over the last 3 decades has resulted in a better understanding of the biochemical process that leads to the production and accumulation of Aβ. More specifically, the proteolytic processing of APP by α-secretase on the cell membrane generates soluble APPα, which has been implicated in neuronal plasticity and synaptogenesis [146]. However, those APP molecules that are not processed by α-secretase are endocytosed and cleaved by β-secretase 1 (BACE1) and γ-secretase to generate Aβ 1–40 and 1–42 peptides [147,148,149], which have a harmful effect on cell survival and synaptic structure and function [150,151]. Inasmuch as our understanding of this process has grown, it has led to the discovery of different therapeutic strategies to minimize Aβ deposition that have been successfully tested in animal models of AD [152], but unsuccessful [153] or only partially effective in AD patients [154].

The last three decades of research on the role of the plasminogen activating system in the pathophysiology of AD have focused almost exclusively on the ability of tPA and uPA to cleave Aβ deposits. However, recent studies have also discovered a role for uPA in the pathogenesis of synaptic dysfunction in AD that does not require the conversion of plasminogen into plasmin.

#### 5.5.1. Plasminogen Activators and the Formation of Aβ Deposits

A role for plasminogen activators in the pathogenesis of AD was suggested by in vitro studies showing that plasmin triggers α-secretase-induced cleavage of Aβ in lipid rafts [155] and cleaves insoluble Aβ fibrils [156,157]. This was followed by work revealing a reduction in the expression and activity of plasmin in AD brains [158], most likely due to a decrease in tPA activity [157]. These observations were contested by a different group of investigators that detected normal concentrations of plasminogen and plasmin in the brains of AD patients [159], and postulated that the reported decrease in plasmin was actually due to the disruption of lipid rafts by abnormal cholesterol metabolism in the neuronal membrane [160].

Most of the studies on the role of plasminogen activators on the formation of Aβ deposits have been performed with tPA. Hence, it has been found that insoluble Aβ activates tPA [161] and increases the expression of tPA mRNA in cerebral cortical neurons, purportedly as an attempt to trigger plasmin-induced cleavage of extracellular insoluble Aβ-containing plaques [158]. In discrepancy with these studies, in vivo studies with mouse models of AD have revealed that chronic elevation of Aβ actually decreases tPA activity by enhancing the inhibitory effect of PAI-1 on tPA, and that the intracerebral injection of Aβ causes neuronal degeneration in animals genetically deficient in either tPA or plasminogen [162].

More specifically, the expression of PAI-1 is increased in the cerebrospinal fluid [163] and the brains of AD patients [164]. The clinical relevance of these observations is underscored by experimental work indicating that the genetic deletion of PAI-1 in the brain of a murine model of AD reduces the deposition of Aβ by increasing tPA-induced plasmin-mediated cleavage of Aβ-containing plaques [164]. Together, these data have led to the proposal of a model in which increased PAI-1 activity in the brain of AD patients abrogates tPA-induced plasmin-triggered cleavage of Aβ deposits. In seeming contradiction with a protective role of tPA in AD, other studies have shown that this plasminogen activator actually mediates the neurotoxic effect of Aβ via ERK ½ activation [165]. The role of uPA on the formation of Aβ deposits has been less well studied. Nevertheless, it has been reported that Aβ increases the abundance of uPA mRNA [156], and that as described for tPA, uPA also induces plasmin-mediated cleavage of insoluble Aβ-containing extracellular plaques [166].

#### 5.5.2. Role of Plasminogen Activators in Synaptic Dysfunction in AD

The idea that the extracellular accumulation of insoluble Aβ peptides is the cause of the cognitive decline observed in AD patients [167] has been challenged by neuropathological and clinical studies indicating that the development of cognitive deficits in these individuals correlates with abnormalities in synaptic structure and function more than with the number of tangles and insoluble Aβ-containing plaques [168,169]. This concept is of significant translational importance, because synaptic dysfunction is an early event in the pathogenesis of AD that is amenable to therapeutic interventions to prevent its development.

Our knowledge of the synaptic role of Aβ has increased significantly during the last 30 years. Hence, we know that the production of Aβ increases during neuronal activity [150], and that while at low concentrations, soluble Aβ induces presynaptic facilitation, at high concentrations triggers post-synaptic depression [170] by decreasing the abundance of glutamatergic receptors in the postsynaptic density [161,171] and enhances the excitotoxic effect of glutamate by blocking its reuptake from the synaptic cleft [172]. Additionally, high concentrations of Aβ impair long-term potentiation (LTP) and enhance long-term depression (LTD) by blocking α-amino-3-hydroxy-5-methyl-4-isoxazolepropionic acid (AMPA) and NMDA receptor function [173], and augment the excitotoxic effect of glutamate by blocking its reuptake from the synaptic cleft [172]. Importantly, increased soluble Aβ has been linked to synaptic depression and the disruption of neuronal network activity in the brains of AD patients [171]. Furthermore, it has been reported that uPA antagonizes the harmful effect of Aβ on synaptic structure and function by a mechanism independent of its ability to trigger proteolytic cleavage of Aβ-containing plaques [68].

Recent studies have shown that cleavage of Aβ-containing plaques is not the only role of uPA in AD brains. Hence, it has been found that the expression of uPA, but not of its receptor (uPAR), is decreased in the synapses of AD patients and 5XFAD mice (express human APP with the Swedish (KM670/671NL), Florida (I716V) and London (V717I) mutations together with a mutant presenilin 1 (M146L, L286V) under the control of the murine Thy-1 promoter), by the ability of Aβ to halt the transcription of uPA mRNA in neurons but not in astrocytes [68]. The translational importance of these findings is supported by observations indicating that treatment with recombinant uPA abrogates the harmful effect of soluble Aβ on synaptic structure and function, via its ability to induce the expression of neuronal cadherin (NCAD). Remarkably, in contrast with the reported effect of tPA and uPA on the proteolytic cleavage of Aβ-containing plaques, the effect of uPA on the synapses of AD patients and animal models of AD does not require the generation of plasmin.

#### 5.5.3. Plasminogen Activators, Physical Activity and Alzheimer’s Disease

Physical activity has a direct effect on the expression and activity of components of the plasminogen activating system. More specifically, 6 months of intensive physical activity increase the intravascular concentration of tPA and uPA, and this effect is accompanied by a substantial decrease in the levels of PAI-1 [15,174]. These observations are of significant importance when interpreted in the context of studies showing that physical activity decreases the risk of AD [175] and improves the attention span, memory and executive functioning of healthy individuals [176]. Thus, it is tempting to postulate that plasminogen activators mediate the protective effect of exercise on cognitive function and the risk of developing AD. However, although scientifically plausible, to this date there are no data to support this hypothesis. 

### 5.6. Plasminogen Activators in Parkinson’s Disease 

Parkinsonism is a clinical syndrome characterized by bradykinesia, resting tremor, rigidity and postural and gait impairment. Most cases of parkinsonism are caused by Parkinson’s disease (PD), which is a neurodegenerative disease that affects 3% of the population older than 65 years of age [177]. The neuropathological hallmarks of this disease are the loss of dopamine-containing neurons in the substantia nigra and the presence of α-synuclein-containing intracellular inclusions. The extracellular levels of α-synuclein depend not only on its release from neurons, but also on its removal by proteolytic degradation. It is unclear if α-synuclein induces the expression or activity of tPA or uPA. However, plasmin cleaves and degrades α-synuclein, and α-synuclein upregulates PAI-1 [178]. It has thus been proposed that an excess of PAI-1 in the brains of PD patients prevents plasmin-induced clearance of α-synuclein aggregates [179]. The translational relevance of these findings is supported by the fact that increased levels of PAI-1 have been linked to a worse clinical prognosis in PD patients [180]. Despite the importance of these observations, to this date it is unclear if tPA- or uPA-catalyzed plasmin generation plays a role in the pathogenesis of this disease.

### 5.7. Plasminogen Activators in Amyotrophic Lateral Sclerosis (ALS)

Amyotrophic lateral sclerosis (ALS) is a motor neuron disease characterized by a progressive decline in motor function caused by weakness and spasticity without sensory loss. Knowledge on the role of plasminogen activators in the pathogenesis of ALS is still in its earlier stages. However, it has been reported that plasminogen from ALS patients, and recombinant tPA and plasmin, induce motoneuron degeneration in BALB/c mice [181]. Likewise, experimental work with G93 mice (with a SOD1 mutation linked to familial ALS) and samples from ALS patients revealed that the abundance of uPAR increased in the ventral horn of the spinal cord of ALS patients and G93 mice, along with enhanced uPA-dependent plasminogen activation in advanced stages of this disease. Remarkably, treatment with an inhibitor of uPA prolonged survival in these animals [182].

## Data Availability

Not Applicable.
